# *Neurofilament Heavy polypeptide* CpG island methylation associates with prognosis of renal cell carcinoma and prediction of antivascular endothelial growth factor therapy response

**DOI:** 10.1002/cam4.181

**Published:** 2014-01-27

**Authors:** Natalia Dubrowinskaja, Kai Gebauer, Inga Peters, Jörg Hennenlotter, Mahmoud Abbas, Ralph Scherer, Hossein Tezval, Axel S Merseburger, Arnulf Stenzl, Viktor Grünwald, Markus A Kuczyk, Jürgen Serth

**Affiliations:** 1 Department of Urology, Hannover Medical School30625, Hannover, Germany; 2 Department of Urology, Eberhard-Karls University72074, Tübingen, Germany; 3 Department of Pathology, Hannover Medical School30625, Hannover, Germany; 4 Department of Biometry, Hannover Medical School30625, Hannover, Germany; 5 Department of Hematology, Hemostasis, Oncology and Stem Cell Transplantation, Hannover Medical School30625, Hannover, Germany

**Keywords:** Methylation, risk assessment, translational research, urological oncology

## Abstract

Neurofilament Heavy polypeptid (*NEFH*) belongs to the group of type IV intermediate filament proteins. DNA methylation of the *NEFH* promoter and loss of expression have previously been shown to activate the AKT/β-catenin pathway in tumor cells. When identifying hypermethylation of the *NEFH* CpG island (CGI) in renal cell cancer (RCC) we asked whether methylation could provide clinical or prognostic information for RCC and/or predict therapy response in patients with metastatic RCC (mRCC) undergoing antiangiogenic therapy. Relative methylation of the *NEFH* CGI was analyzed in 132 RCC samples and 83 paired normal tissues using quantitative methylation-specific PCR. Results were statistically compared with tumor histology, clinicopathological parameters, progression-free survival (PFS) as well as with overall survival (OS) in a subset of 18 mRCC patients following antiangiogenic therapy regimens. The *NEFH* CGI methylation demonstrated a tumor-specific increase (*P *<* *0.001), association with advanced disease (*P *<* *0.001), and distant metastasis (*P *=* *0.005). Higher relative methylation was also significantly associated with a poor PFS (HR = 8.6, *P *<* *0.001) independent from the covariates age, gender, diameter of tumors, state of advanced disease, and local and distant metastasis. Median OS following targeted therapy was 29.8 months for patients with low methylation versus 9.8 months for the group with high methylation (*P *=* *0.028). We identified *NEFH* methylation as a candidate epigenetic marker for prognosis of RCC patients as well as prediction of anti-vascular endothelial growth factor-based therapy response.

## Introduction

Renal cell cancer (RCC) is among the 10 most frequent causes of cancer death of men in western countries 1. Surgical treatments as nephrectomy or partial nephron-sparing resection represent the standard therapy of localized and locally advanced RCC. Although improvements of overall survival (OS) due to anti-vascular endothelial growth factor (VEGF)-based therapies have been achieved, patients with advanced disease still have a poor prognosis 2,3. Moreover, patient stratification for clinical trials is limited to the use of clinically based scorings such as the Memorial Sloane Kettering Cancer Center (MSKCC) or the Heng systems 4,5. In view of limitations of current prognostic models it has been argued that biologically based markers could improve both the quality of prognostic information and prediction of therapy response 6. Moreover, new biologically based markers could identify, as a side effect, new molecular targets in signal transduction of RCC pivotal for progression of the disease 6.

Clear-cell renal cell carcinoma (ccRCC) is the most frequent histological entity of all RCCs. The question for the molecular architecture of changes underlying this tumor has not been fully answered at yet. Mutations in the von Hippel-Lindau (*VHL*) gene have been found in 52–83% of ccRCCs patients^7^–^9^ and changes in the polybromo 1 (*PBRM1*) gene have been reported as the second most common mutation detectable in 23–54% of ccRCCs^9^–^11^ while other mutations such as of the SET domain containing 2 (*SETD2)* and BRCA1-associated protein-1 (*BAP1)* genes exhibited only low occurrence 8,9.

Therefore, rather few common mutations have been revealed to be associated with ccRCC despite exome and genome-wide sequencing analyses of large patient cohorts thus far 9,10.

Statistical association of somatic mutations with adverse clinical parameters such as higher nuclear grade, necrosis and advanced stage along with evidence for a relationship with poor survival of patients have only been reported for the *BAP1* gene 11. Consistently, The Cancer Genome Atlas network (TCGA) solely identified mutations in the *BAP1* gene to be associated with a worse survival of patients 9. Of note, the TCGA study also showed that a great variety of overall rarely observed genetic alterations including mutations and gains and losses of sequences were found to be individually combined in tumors thus restraining the identification of simple functional conclusions as well as of statistical relationships such as the clinical outcome of patients 9.

On the other hand, many epigenetic DNA-methylation-based alterations have already been reported to occur with a high frequency in ccRCC 9,12–15 and to show high odds ratios for adverse clinical or pathological parameters 14,16–20. Moreover, a subgroup of these methylation markers demonstrated independence from important clinical parameters, such as stage, grade, diameter of tumor, and status of local or distant metastasis 14,16,18,20,21. Interestingly, the most frequent common gene mutations detected so far in ccRCC were either functionally related to histone modification and stabilization, thus mechanisms indented with expression states of genes and DNA methylation 22, or, as in case of *VHL*, were demonstrated to cause an increase in epigenetic alterations including DNA methylation 23. Consequently, epigenetic alterations could be a hallmark of RCC and might be an important mean for molecular-based prognosis or prediction of this disease.

In the course of a combined in silico analysis of gene expression and a genome-wide re-expression analysis using the demethylation agent 5-aza-2′-deoxycytidine in renal cancer cell lines we already identified new potential DNA-methylation-based candidate prognosticators for ccRCC 19,20 and also found the Neurofilament Heavy polypeptid (*NEFH*) CGI as a further epigenetic mark of potential interest. *NEFH* is located on chromosome 22q12.2, encodes for a 200 kDa protein and is classified to the group of type IV intermediate filaments which are important components of the neuronal cytoskeleton 24. It has been reported that tumor-specific loss of *NEFH* mRNA expression occurs in prostate carcinoma 25. Furthermore, higher CGI methylation has been detected in normal esophageal mucosa cells of smokers, indicating the presence of premalignant epigenetic alterations in precancerous lesions as a cancer risk factor 26. Moreover, *NEFH* promoter methylation in esophageal squamous cell carcinoma (ESCC) has been functionally linked with loss of expression and activation of the *v-akt* murine thymoma viral oncogene homolog (AKT)/β-catenin pathway also leading to increased glycolysis rates and changes in mitochondria 27.

Here we identified a *NEFH* methylation marker that shows specific hypermethylation in RCC and is significantly associated with adverse clinicopathological parameters of the tumor as well as progression-free survival (PFS) of RCC patients. Moreover, *NEFH* methylation associates with OS of patients with metastatic disease undergoing targeted therapy regimes. This study suggests *NEFH* methylation both as an independent prognosticator and predictor for patients with ccRCC and metastatic disease (mRCC).

## Material and Methods

### Study design and patients

Cross-sectional and prognostic analyses were carried out on 114 RCC fresh frozen samples and 83 corresponding histologically normal appearing samples (Table 1) as described previously 20. Survival analyses for mRCC following anti-VEGF-based therapy was done using a cohort of 18 formalin-fixed and paraffin-embedded (FFPE) samples (Table 2). Sample collection was approved by the local ethics committee and informed consent was obtained from each patient. TNM classification was evaluated according to the Union for International Cancer Control 2002 classification as described before 28. Localized and locally advanced RCC describe tumors with pT ≤ 3, lymph node (N) and metastasis (M) negative (N0, M0). Advanced tumors are pT = 4 and/or lymph node positive (N+) and/or positive for distant metastasis (M+). The histological grading was assessed according to Thoenes et al. 29. The time from primary surgery to the time of the first progressive event including local recurrence or a new metastatic site detected by computer tomography scan was designated as PFS independent from the initial TNM status. OS was the period of the first day of systemic therapy until patient's death or the last day of follow-up.

**Table 1:** Patient characteristics.

	All RCC	%	All RCC survival group	%
Total cases	114		50	
Histology				
ccRCC	82	71.9	39	78
papRCC	24	20.2	10	20
Mixed histology	4	3.5	1	2
Not class.	4	3.5	0	
Gender				
Female	40	35	19	38.0
Male	74	65	31	62.0
Age, median (years)	65		66.5	
Distant metastasis				
M0	88	77.2	41	82.0
M+	26	22.8	9	18.0
Lymph node metastasis				
N0	100	87.7	47	94.0
N+	14	12.3	3	6.0
T-classification				
pT1	11	9.6	1	2.0
pT1a	34	29.8	20	40.0
pT1b	19	16.7	9	18.0
pT2	7	6.1	3	6
pT3	5	4.4	2	4
pT3a	9	7.9	2	4
pT3b/c	24	21.1	12	24
pT4	1	0.9	0	
NA	4	0.9	1	2.0
Differentiation				
G1	22	19.3	6	12.0
G1–2	15	13.2	8	16.0
G2	58	50.9	28	56.0
G2–G3	8	7.0	3	6.0
G3	11	9.5	5	10.0
State of disease				
Loc./Loc.Adv.Disease^1^	81	71.1	39	78.0
Adv. Disease^2^	32	28.1	11	22.0
NA	1	0.9	0	
Paired samples				
All RCC	83			
ccRCC	63			

ccRCC, clear cell renal carcinoma; papRCC, papillary renal cell carcinoma.

1 pT ≤ 3, N0, M0.

2 pT = 4 and/or N+ or M+.

**Table 2:** Characteristics of patients with mRCC undergoing anti-VEGF-based therapy.

	n (%)
Total cases	18 (100)
Histology	
ccRCC	16 (89)
Papillary	1 (6)
Chromophobe	1 (6)
Gender	
Female	7 (39)
Male	11 (61)
Distant metastasis^1^	
M0	0
M+	3 (17)
Mx	15 (83)
Lymph node metastasis^1^	
N0	2 (11)
N1	1 (6)
N2	1 (6)
Nx	14 (78)
T-classification^1^	
1	1 (6)
1a	2 (11)
1b	2 (11)
2a	2 (11)
3b	6 (33)
4	2 (11)
x	3 (17)
Differentiation	
G1	0 (0)
G2	12 (67)
G3	6 (33)
TKI-first line	
Sunitinib	12 (67)
Sorafenib	4 (22)
Bevacizumab	1 (6)
Axitinib	1 (6)
Death within follow-up	
No	4 (22)
Yes	14 (78)
Age, median, min-max (y)	59.5 (48–80)
OS, median, min-max (m)	12.4 (0.8–59.3)

ccRCC, clear cell renal carcinoma; TKI, tyrosine kinase inhibitor; OS, overall survival; mRCC, metastatic renal cell carcinoma.

* TNM status refers to the initial histopathological evaluation after nephrectomy.

### Cell lines

Human tumor cell lines and primary cells were short-term cultured immediately following purchase and identity control by the manufacturer (Cell line services, Heidelberg, Germany; Lonza, Basel, Switzerland) exclusively for the purpose of DNA isolation as described previously 19,20.

### DNA isolation, bisulfite conversion of DNA, and quality measurements

Isolation of DNA from frozen sections and histopathological evaluation of control sections for estimation of tumor cell content were performed as described before 20. FFPE samples were punched out as cylinders of approximately 2 mm height and 1.5 mm diameter by the pathologist following examination for histopathology and tumor cell content. DNA was isolated using an automated MagNA Pure LC system (Roche Diagnostics Deutschland, Roche Applied Science, Mannheim, Germany). Extracted DNA was characterized for yield, purity, and length distribution by the use of spectralphotometry and agarose gel electrophoresis and then subjected to bisulfite conversion using the EZ DNA Methylation-Gold™ Kit (Zymo Research Corporation, Irvine CA, USA). Yield and degree of conversion of converted DNA was controlled by independent measurements of a repetitive sequence ALU-C4 (QC1) and a single copy *β*-Actin (*ACTB*) sequence (QC-2) as described before.^20^ Fully methylated and converted control DNA (M) as well as unmethylated bisulfite-converted control DNA (U) were prepared as described previously 20,30.

### Quantitative methylation-specific PCR (qMSP) analysis

Quantitation of *NEFH* methylation was carried out by a quantitative real-time fluorimetric 5′ exonuclease PCR assay. The qMSP primers 5′-ACCCGACCGCGACGACTATA-3′ (forward), 5′-CGTCGAAGTTTATTATGGTTTGAGTAGG-3′ (reverse) and the Taqman® probe 5′-FAM-CGCCCTAATACTACCGCAATACCTCCCGC-BHQ-3 were created by use of the Beacon Designer™ software (PREMIER Biosoft, Palo Alto CA, USA). The qMSP analysis covered nine CpG sites on chromosome 22 at positions 29,876,165, ∼169, ∼171, ∼174, ∼206, ∼218, ∼231, ∼263, and ∼266 according to the GRCh37/hg19 annotation in the UCSC genome browser 31,32. Real-time PCRs were performed as duplicates on an ABI 7900HT (Life technologies, Foster City, CA, USA) in 384-well plates using an automated liquid handling system as described previously 20. The experimenters carried out measurements without knowledge of type, order, clinicopathological or survival status of samples. Relative methylation levels were calculated as an analogue of the delta–delta *C*_t_ method by normalizing the difference of *NEFH* methylation-specific real-time detection and methylation independent internal QC1 control measurement with the corresponding difference for the fully methylated DNA control samples as described previously 20.

### Statistical analyses

Explorative statistical data analyses were performed using the statistical software R 2.15 33. *P < *0.05 was considered as significant. Relative methylation levels were transformed into the natural logarithmic scale before conducting further statistical calculations. Linearity and PCR efficiency of the qMSP assay were analyzed using linear regression analysis. Mean relative methylation levels observed for paired tumor and adjacent normal appearing tissues were compared using the two-sided *t*-test for paired data. Independent tissue sample groups were compared using univariate logistic regression analysis. Dichotomization of methylation levels for analysis of PFS were performed using the R package “maxstat” providing calculation of the optimum threshold. Analysis of PFS was carried out using univariate Cox regression analysis. Thus, *P*-values and hazard ratios (HR) could be calculated for comparison with results of bivariate analyses considering covariates. OS of patients undergoing targeted therapy was calculated using log Rank statistics.

## Results

### Measurement of *NEFH* CGI methylation in technical controls, normal primary cells, and tumor cell lines

**Figure 1:**
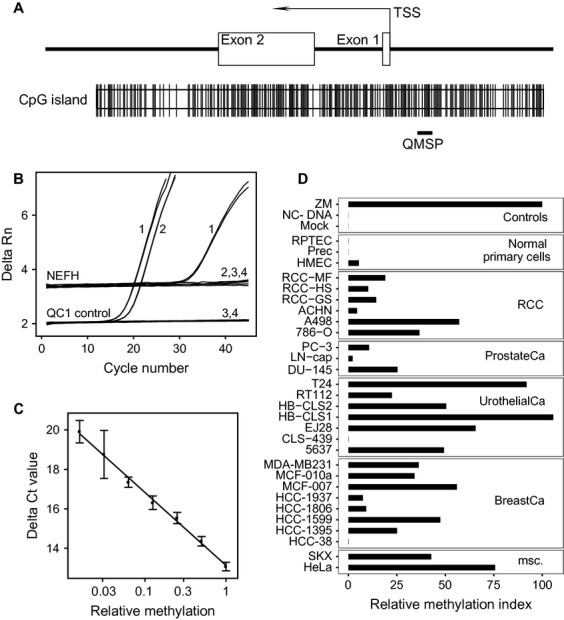
(A) Neurofilament Heavy polypeptid (*NEFH*)–CpG island (CGI) organization and location of the qMSP assay relative to the transcription start site (TSS) and exon 1 and 2. CpG sites are indicated by vertical lines. (B) qMSP data for control measurements in duplicate of fully methylated DNA (1), unmethylated DNA (2), unconverted DNA (3) and the blank sample (4). (C) Measurement of assay linearity and efficiency using a twofold dilution series of fully methylated in non-methylated control DNA. (D) Relative methylation levels determined by qMSP for control samples, normal primary cells and various cancer cell lines representing human cancers as specified.

The analysis of converted methylated (M), converted non-methylated (U), and non-converted DNA control samples demonstrated that the *NEFH* qMSP specifically detects M–DNA while U and non-converted samples remained undeterminable both in the QC1 control reaction as well as in the *NEFH*-specific PCR (*C*_t_ > 45 cycles, Figure 1B). A log2 dilution series adjusted for a constant total amount of converted DNA showed good efficiency and high linearity of the qMSP assay (Figure 1C). Using the slope of the qMSP calibration line we calculated a PCR efficiency of 0.95. Measurement of methylation in cell lines used as substitutes for frequent human cancers as well as normal primary cells and control DNAs demonstrated no methylation neither in normal primary cells of renal or prostatic origin nor in mock controls (Figure 1D). Low methylation was detected for normal mammary primary cells. Breast and urothelial cancer cell lines overall demonstrated highest relative methylation values while >25% relative methylation was detected in two of six RCC and one of three prostate cancer cell lines (Figure 1D).

### The *NEFH* CGI shows hypermethylation in RCC

**Figure 2:**
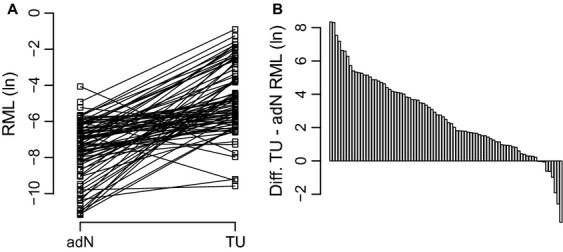
Tumor-specific hypermethylation of the Neurofilament Heavy polypeptid (*NEFH*)–CpG island (CGI) sub-region analyzed. (A) Pairwise comparison of relative methylation levels (RML) in natural logarithmic scale for adjacent normal appearing (adN) and paired tumor tissue (TU) samples (B) Assorted differences of RML observed in adN and TU samples.

The comparison of relative methylation as observed in paired tumor (TU) and adjacent normal (adN) tissues revealed the presence of a tumor-specific hypermethylation (*P *<* *0.001). Nearly all of the paired TU versus adN comparisons demonstrated a substantial increase in tumor-specific methylation (Fig. 2A and B). The median relative methylation values found in adN and TU tissues corresponded to 0.087% and 0.634% indicating a 7.3-fold average increase in highly methylated sequences in tumors. *NEFH* methylation of normal and cancer tissues were not correlated (*P *=* *0.65, *r* = 0.05, Pearson′s correlation analysis).

### *NEFH* methylation and association with clinicopathological parameters

**Figure 3:**
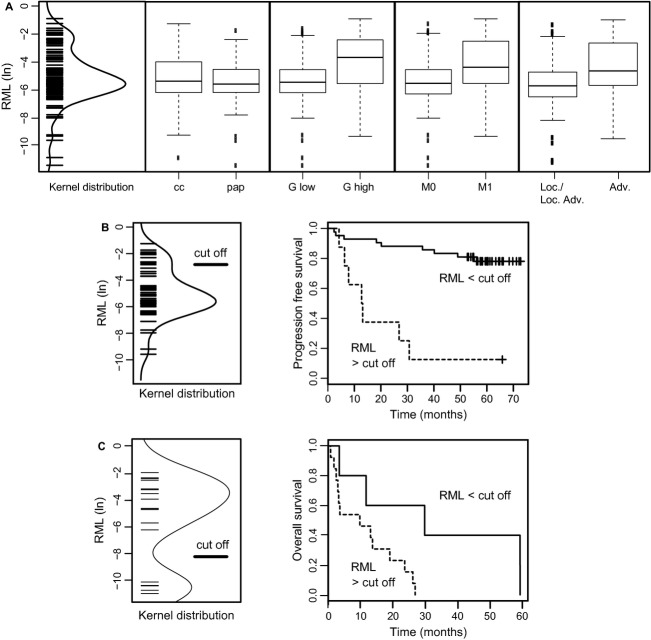
(A) Distribution with kernel density estimation for relative methylation levels (RML) in natural logarithmic scale as detected in all tumor samples (left panel). Box plot analyses showing tumor subset-specific relative methylation levels for clear cell (cc) or papillary (pap) histology, low-(*G* ≤ 2) or high-grade (*G* > 2) tumors, negative (M0) or metastasis-positive (M+) patients and localized or locally advanced (pT ≤ 3, N0, M0) or advanced (pT > 3 and/or N+, M+) disease (B) Distribution of relative methylation values in the survival analysis group with kernel distribution and indication of the statistically optimized cutoff value of RML of 5.9% (−2.85 in the natural logarithmic scale, left panel). Kaplan–Meier plot analysis illustrating relative progression-free survival of all renal cell cancer (RCC) patients following dichotomization (right panel). (C) Distribution and cutoff level of relative methylation levels observed for the therapy group (left panel) and Kaplan–Meier plot analysis (right panel) showing relative overall survival of patients under targeted therapies for treatment of advanced disease.

We found that higher methylation of the *NEFH* sub-region analyzed was statistically associated with the status of distant metastasis, advanced disease, and high-grade tumors (*P *=* *0.005, OR 1.46 [1.12–1.91, 95%CI]; *P *<* *0.001, OR 1.56 [1.20–2.03, 95%CI]; *P *=* *0.012, OR 1.46 [1.09–1.95, 95%CI], Figure 3A, Table 3A). Age, gender, diameter of tumors, and lymph node status were not related with higher methylation in tumors. No significant difference was found between tumors of clear cell and papillary histology (*P *=* *0.270, OR 0.87 [0.68–1.11, 95%CI]). Thus, all subsequent statistical analyses were carried out without consideration of tumor histology.

**Table 3:** Statistical association between *NEFH* CGI methylation and clinicopathology of patients with RCC; (A) Univariate logistic regression analysis for tumor group comparisons; (B) Univariate analysis of progression-free survival using Cox regression; (C) Bivariate analysis of progression-free survival using Cox regression.

(A)						
Parameter of dichotomization	Median RML^1^	Median RML^1^	*P*-value^2^	OR	95% CI	Adjusted *P-*value^2^
Dist. metastasis (M0/M +)	−5.5	−4.3	0.005	1.46	1.12–1.91	0.019
Lymph node met. (N0/N +)	−5.4	−5.0	0.202	1.22	0.9–1.66	0.362
Grade (low/high)	−5.4	−3.6	0.012	1.46	1.09–1.95	0.035
Diameter^3^	−5.3	−5.5	0.178	1.19	0.93–1.52	0.362
Loc.&loc.adv.dis./adv. dis.	−5.6	−4.5	<0.001	1.56	1.20–2.03	0.005

(B)			
Variate	*P*-value^4^	HR	95% CI
*NEFH* methylation	<0.001	8.61	3.03–24.5
Metastasis	<0.001	5.88	2.17–15.9
Lymph node status	0.283	2.26	0.51–10.0
Loc.&loc.adv.dis./adv. dis.	<0.001	5.33	1.99–14.3
Gender	0.187	2.14	0.69–24.5
Age^5^	0.560	0.75	0.28–2.00
Diameter^5^	0.200	2.23	0.65–7.63

(C)			
Variate/Covariate	*P*-value^6^	HR	95% CI
*NEHF* methylation	<0.001	11	3.53–34.2
Metastasis	<0.001	7.27	2.52–20.9
*NEHF* methylation	<0.001	6.96	2.35–20.7
Loc &loc.adv.disease/adv. disease	0.005	4.21	1.53–11.6
*NEHF* methylation	<0.001	7.88	2.75–22.6
Gender	0.357	1.72	0.54–5.42
*NEHF* methylation	<0.001	17.1	4.62–63.3
Age^5^	0.043	0.28	0.08–0.96
*NEHF* methylation	0.022	5.07	1.26–20.4
Diameter^5^	0.368	1.79	0.50–6.38

CI, confidence interval; HR, hazard ratio; OR, odds ratio; RML, Relative methylation level (natural logarithmic scale); CGI, CpG Island; RCC, renal cell carcinoma.

1 Median relative methylation level (RML) of dichotomized groups.

2 Univariate logistic regression, correction for multiple testing by Holms.

3 Localized or locally advanced (T ≤ 3. N0, M0) and advanced disease (pT = 4 and/or N +, M+).

4 Univariate Cox regression analysis.

5 Dichotomized using median of parameter.

6 Bivariate Cox regression analysis.

### *NEFH* methylation is independently associated with decreased PFS of patients

Patients measured with methylation higher than the statistically calculated cutoff value of 5.9% relative methylation showed a significantly shortened PFS (*P *<* *0.001, HR = 8.61 [3.03–24.5, 95%CI], Table 3B). The corresponding Kaplan–Meier plot shows that six out of seven (86%) patients with higher methylation were found with disease progression within 30 months (Figure 3B). In contrast 80% of tumors identified with low methylation did not show disease progression within 70 months of follow-up. High HRs were not only observed in univariate but also in pairwise bivariate Cox regression analyses considering status of distant and lymph node metastasis, state of localized or locally advanced and advanced disease, gender, age, tumor diameter, and tumor differentiation as covariates. Bivariate HRs ranged between 5.1 and 17.1 and for the parameter methylation significance was observed in all bivariate statistical evaluations (*P *<* *0.001–*P *=* *0.022, Table 3C).

### *NEFH* methylation is associated with OS of patients undergoing antiangiogenic therapy

The analysis of *NEFH* methylation in tumor samples of patients undergoing targeted therapy for treatment of mRCC revealed a bimodal distribution of methylation values identifying low-and high-methylated tissue groups making a statistical calculation of a cutoff value for dichotomization redundant (Fig. 3C). To evaluate whether both groups differ with respect to the OS of patients we performed a Kaplan–Meier survival analysis and log-rank statistics (Fig. 3C). We found a median OS of 29.8 (11.7-NE) months for patients with low methylation in tumor tissues while patients with higher methylation demonstrated a mean OS of 9.8 (3.0-NE) months (*P *=* *0.028). Using a cutoff of 6 months for PFS as recently suggested as prognosticator of OS 34, analysis of *NEFH* methylation allows detection of therapy failure with a sensitivity of 0.91 (0.62–0.98, 95% CI).

## Discussion

Our methylation analyses showed that higher relative methylation levels of distinct CpG sites within the *NEFH* CGI are statistically related with unfavorable clinical and pathological characteristics of RCCs such as presence of distant metastasis, state of advanced disease as well as poor differentiation of tumor cells. Hence, *NEFH* appears as a candidate for functional alterations occurring in aggressive RCCs. This understanding is sustained by our finding that higher *NEFH* methylation is associated both with PFS of patients of the prognosis cohort as well as OS of the patients undergoing anti-VEGF-based therapy. Interestingly, we found that methylation appeared as a significant variable for PFS independent from all available clinical and pathological confounders including state of local and distant metastasis, states of localized/locally advanced or advanced disease, tumor diameter, grade of tumors, age, and gender. *NEFH* methylation remained as a highly significant factor exhibiting high and notable constant HRs in all bivariate survival analyses suggesting this marker as independent prognosticator for RCC. Thus, this study shows to the best of our knowledge for the first time, that *NEFH* methylation is associated with the survival of RCC patients and is a candidate predictor for the response of multiple sequential-targeted therapies in advanced RCC.

Loss of expression of *NEFH* was first found in prostatic tumors 25, while epigenetic alterations of *NEFH* have initially been reported in the context of “field cancerization” to occur as premalignant DNA-methylation events detectable in normal epithelial cells of tissues at higher tumor risk 26. Subsequently, *NEFH* was functionally identified to exhibit attributes of a tumor suppressor as knockdown experiments revealed increased tumorigenicity in mice while expression of *NEFH* was associated with diminished cell growth and reduced colony formation in vitro 27. Moreover, DNA methylation of *NEFH* associated with high-grade and -stage of ESCC and epigenetic silencing caused activation of the AKT/β-catenin pathway 27, therefore, indicating that epigenetic alterations of *NEFH* could contribute to human carcinogenesis. Considering that *NEFH* methylation or altered expression have not been reported for cancers other than ESCC thus far, our findings of hypermethylation taking place both in RCC as a highly malignant tumor and in tumor cell lines from mammary, prostatic, and urothelial cancers further underline the potential relevance of *NEFH* in carcinogenesis.

The *NEFH* methylation mark may be also of clinical interest because it demonstrated independence from both grade as well as status of distant metastasis, which seems remarkable as both factors represent strong classical clinicopathological parameters for the prognosis of disease progression. It is known that patients undergoing antiangiogenic therapy for treatment of metastatic RCC exhibit different survival characteristics 34. We, therefore, inves-tigated in a pilot study whether *NEFH* methylation is also associated with survival of patients treated for advanced disease and found high-and low-methylated primary tumors showing nearly no overlap between both groups. Increased methylation associated with a strongly shortened median OS and using a cut point of 6 months for PFS as a prognosticator of OS 34, analysis of *NEFH* methylation would allow detection of therapy failure with a sensitivity of 91%. To the best of our knowledge, such a result has not been reported for any DNA-based marker at yet, suggesting this epigenetic mark as a promising candidate molecular prognosticator and predictor for RCC.

This study identified statistical associations between an epigenetic alteration and worse patient survival and therapy response. Thus, this study provides evidence that an unknown functional relationship of NEFH and cellular signaling might exist contributing to the development of aggressive RCC. Targeted therapeutic intervention in RCC aims at specific components of either the AKT/mammalian target of rapamycin (mTOR) or the mitogen-activated protein kinase (MAPK) pathways, together providing hypoxia-related signal transduction in RCC 35. Notably, NEFH shows functional interaction with both pathways. First, knockdown experiments in esophageal cancer cell lines revealed that NEFH is connected with the AKT pathway via *Gsk3ß* 27, and, moreover, it has been reported that AKT/mTOR pathway alterations occur in RCC and affect prognosis of patients 36. Considering that NEFH has been suggested to be functionally associated with altered metabolism of tumor cells such as increased glycolysis via the AKT/β-catenin pathway 27, it can be hypothesized that *NEFH* alterations could contribute to the recently described metabolic shift of aggressive renal cancer cells found as a result of integrative genome-wide analyses of molecular alterations in ccRCC 9.

Second, NEFH is a substrate of the MAP kinases MAPK3 and MAPK1 37 and, phosphorylation of NEFH by stress-activated p38 protein kinases (MAPK11,-12,-14) has been annotated for the amyotrophic lateral sclerosis pathway in Kyoto Encyclopedia of Genes and Genomes indexes 38.

Conclusively, this study identifies increased *NEFH* DNA methylation as a candidate marker for independent prognosis of PFS of RCC patients. Moreover, our results suggest that *NEFH* methylation also predicts OS of metastatic patients undergoing targeted therapies. Our analyses underline the need for a functional analysis of NEFH in RCC and other cancers, possibly contributing for future individualized prognosis and treatment of patients.
